# Competitive organizational climate and artificial intelligence (AI) acceptance: the moderating role of leaders’ power construal

**DOI:** 10.3389/fpsyg.2024.1359164

**Published:** 2024-03-25

**Authors:** Kyriaki Fousiani, Georgios Michelakis, Pieter A. Minnigh, Kiki M. M. De Jonge

**Affiliations:** ^1^Department of Psychology, Organizational Psychology Section, University of Groningen, Groningen, Netherlands; ^2^Independent Researcher, Groningen, Netherlands; ^3^Groeiflow, Groningen, Netherlands

**Keywords:** competitive organizational climate, artificial intelligence (AI) acceptance in organizations, leadership, power construal, power as responsibility, power as opportunity, employee attitudes

## Abstract

**Introduction:**

The incorporation of Artificial Intelligence (AI) in organizations is pivotal to deal with work-related tasks and challenges effectively, yet little is known about the organizational factors that influence AI acceptance (i.e., employee favorable AI attitudes and AI use). To address this limitation in the literature and provide insight into the organizational antecedents influencing AI acceptance, this research investigated the relationship between competitive organizational climate and AI acceptance among employees. Moreover, given the critical role of a leader in employee attitude and behavior, we examined the moderating role of leaders’ power construal as responsibility or as opportunity in this relationship.

**Methods:**

Study 1 was a three-wave field study among employees (*N* = 237, *M*_*age*_ = 38.28) working in various organizations in the UK. The study measured employees’ perception of a competitive organizational climate at Time 1, leaders’ power construal (as perceived by employees) at Time 2, and employee attitudes towards AI and their actual use of AI in the workplace at Times 2 and 3. Study 2 was a 2 (climate: highly competitive vs. low competitive) by 2 (power construal: responsibility vs. opportunity) experiment among employee participants (*N* = 150, *M*_*age*_ = 37.50).

**Results:**

Study 1 demonstrated a positive relationship between competitive climate and employee AI use over time. Furthermore, both studies revealed an interaction between competitive climate and leader’s power construal in the prediction of employee AI acceptance: In Study 1, competitive climate was negatively related to AI acceptance over time when leaders construed power as opportunity. In Study 2 competitive climate was positively related to AI acceptance when leaders construed power as responsibility rather than as opportunity.

**Discussion:**

These results underscore the organizational factors that are required in order for employees to shape favorable attitudes towards AI and actually use AI at work. Importantly, this research expands the limited body of literature on AI integration in organizations.

## Introduction

Artificial intelligence (AI) stands at the forefront of the fourth industrial revolution, where organizations are strategically integrating it as a vital tool to address a diverse range of daily management and work-related challenges (Schwab, 2017; Syam and Sharma, 2018). AI use offers benefits to employees as AI encompasses the capability of machines to carry out cognitive functions traditionally associated with human thinking, such as learning, interaction, problem-solving, creativity and innovation (Wamba-Taguimdje et al., 2020; Raisch and Krakowski, 2021; De Jonge et al., in press). Eventually, AI use helps employees to better observe, reason, and adapt to the ever-evolving work environment (Hughes et al., 2019). Importantly, AI seems to complement human intelligence, leading to improvements in quality, accuracy, and precision throughout employees’ tasks (Wilkens, 2020) and provide enormous potential for workplace creativity (De Jonge et al., in press). Besides the benefits of AI for employees, AI offers benefits for organizations as well, as it streamlines manufacturing, enhances decision-making, and improves operational efficiency in businesses (Wright and Schultz, 2018; Kim and Heo, 2022). For instance, AI-driven healthcare robots can monitor patient health (Broadbent et al., 2016); In the retail industry, AI aids inventory management, as seen with Amazon (Kaplan and Haenlein, 2019); In the hotel industry, AI chatbots manage customer stays and routine queries (Chung et al., 2020) and enhance customer service in contact centers (Kirkpatrick, 2017). In product development, AI software can steer the generation and development of new and innovative products (De Jonge et al., in press). Thus, AI acceptance becomes essential for both employees and organizations as it gives them a competitive advantage (Oliveira and Martins, 2011).

Yet, despite the advantages of AI (for a review, see Enholm et al., 2022) employees frequently perceive AI as a double-edged sword with unintended consequences (Wilkens, 2020). Notably, employees often feel uncertain about AI, and its adoption has been linked to increased resignation rates (Brougham and Haar, 2018; Li et al., 2019). Indeed, previous literature indicates that employees worry that advanced systems like AI not only replace human workers, potentially leading to job loss (McClure, 2018), as they can surpass human performance standards (Chui et al., 2015), but also become difficult to control (Fast and Horvitz, 2017). Consequently, employees view AI as a threat they need to fight rather than an opportunity to embrace in their professional lives (Bhargava et al., 2021). A recent review underscores the significance of some key factors that may determine the rejection or acceptance of AI in the workplace, such as employee characteristics and individual differences, as well as organizational factors (e.g., organizational climate and management; for a review, see Yu et al., 2023). While prior research has largely explored the impact of employee characteristics and demographics on AI acceptance and AI use (McClure, 2018; Schepman and Rodway, 2023), little is known about the influence of organizational factors in the acceptance and adoption of AI. To address this gap, this study investigates how two main organizational factors, namely organizational climate and leadership (Yu et al., 2023), influence AI acceptance, specifically, favorable attitudes towards AI and actual use of AI at work.

Organizational climate plays a crucial role in the acceptance of AI as it provides insight into the broader context in which AI technologies are introduced and adopted in a workplace. The organizational climate encompasses various aspects, such as the overall atmosphere, culture, and relationships (Nerstad et al., 2013), which can significantly impact how employees perceive and interact with AI (Yu et al., 2023). For instance, organizational climates fostering innovation and a willingness to explore new ideas are more likely to embrace AI technologies (Mikalef and Gupta, 2021) and enable employees to identify novel AI application opportunities (e.g., Vinchon et al., 2023). Such an organizational climate where employees are motivated to excel by innovating to complete their tasks, is the competitive organizational climate (also referred to as performance climate). Indeed, in highly competitive organizational climates, employees are more motivated to excel by seeking creative and innovative approaches to complete their tasks, especially when their creative efforts are recognized and commended by others (Schrock et al., 2016; Ye et al., 2020). Accordingly, in this study we argue that employees will be more likely to develop favorable attitudes towards AI and to adopt AI for their work-related tasks in a competitive organizational climate, a climate where employees are driven to excel and outperform their colleagues and where they “perceive that organizational rewards are provided contingent on how they perform compared to their peers” (Brown et al., 1998, p. 89; see also Nerstad et al., 2013).

Nevertheless, it is noteworthy that organizational climate alone is not a consistent predictor of employee AI acceptance and its effect may depend on other factors as well. Indeed, as mentioned above, competitive organizational climate encourages employees to demonstrate creativity and innovation –and thus would foster AI acceptance– under the premise that employee efforts and outcomes are being recognized and appreciated (Ye et al., 2020). However, this should not necessarily be the case when employees do not experience such recognition and appreciation. In fact, competitive climates are often experienced as threatening to employees because leaders systematically compare their performances to other coworkers. Moreover, employees feel more easily replaceable and not always rewarded for the extra mile that they take in order to meet the high-performance standards of such competitive climates (Kohn, 1992; Clark et al., 2016). In this light, a competitive climate could be associated with a negative stance towards AI, since AI might be perceived as an additional stressor that intensifies concerns about being replaced and undervalued within such climates (see Li et al., 2019). Indeed, AI acceptance seems to be more likely in environments and contexts that besides creativity and innovation, encourage employee sense of psychological safety and security. When employees feel that they are not being treated with respect, transparency, and care, they tend to develop negative attitudes towards AI and resist its integration (Schepman and Rodway, 2023).

**Figure 1:**
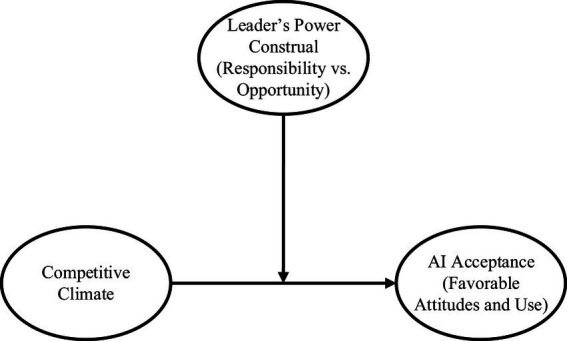
Hypothesized model.

Considering the literature presented above, we argue that the relationship between competitive climate and AI acceptance is contingent on additional organizational factors that can establish a sense of psychological safety and security among employees. Leadership is one of the key factors to consider in this context, as leadership plays a crucial role in shaping the perceptions and experiences of employees within a competitive climate (Fousiani and Wisse, 2022). Indeed, when leaders engage in behaviors that prioritize the goals and interests of their employees, fostering a caring and supportive environment (Sassenberg et al., 2012; Scheepers et al., 2012), employees tend to perceive competitive climates in a more favorable light. In contrast, when leaders prioritize their own self-interests and neglect the needs of their employees (Sassenberg et al., 2012; Scheepers et al., 2012), employees view competitive climates negatively (Fousiani and Wisse, 2022). Building upon this, we propose that the impact of a competitive climate on employees’ favorable attitudes towards AI and their willingness to adopt it is contingent upon leaders’ treatment of employees. Specifically, we hypothesize that a competitive climate will have a positive relationship with employees’ favorable attitudes towards AI and AI use only when leaders deploy a responsible leadership approach towards employees (i.e., care about their employees; De Wit et al., 2017). Such an approach should encourage employees to embrace the challenges and advantages that competitive climates offer while feeling psychologically safe. Conversely, when leaders adopt a self-serving and opportunistic approach, the effect of a competitive climate on AI attitudes and acceptance is expected to be negative. In this case, employees are more likely to perceive the competitive climate as exploitative and self-threatening and may not leverage the potential benefits of AI for creativity and innovation. See Figure 1 for a graphical representation of the hypothesized research model.

This study has several aims. The primary objective is to address a significant gap by examining the impact of organizational factors, such as organizational climate and leadership, on employee attitudes towards AI and their AI use. More specifically, this study emphasizes the role of a competitive organizational climate (Brown et al., 1998, p. 89; Nerstad et al., 2013) where employees are motivated to excel and innovate (Mikalef and Gupta, 2021), as a key driver in fostering favorable attitudes towards and actual use of AI. Moreover, the study introduces the critical concept of leadership (i.e., responsibility or opportunity-based power of the leader; Sassenberg et al., 2014; De Wit et al., 2017) as a moderating factor, that can either enhance or diminish the impact of a competitive climate on AI attitude and use. These contributions offer valuable insights into the intricate connection between organizational factors and AI acceptance (Yu et al., 2023), enhancing our understanding of the dynamics involved in the rapidly evolving landscape of AI adoption in the workplace.

## Literature review

### Antecedents of AI attitude and use and the role of competitive climate

In recent times, AI has captured considerable attention (Yu et al., 2023), primarily propelled by the notable advancements in computer hardware, network speeds, the vast reservoir of accessible data, and the evolution of processing algorithms (Alsheibani et al., 2018). Indeed, given its various benefits both for organizations and employees (for a review, see Enholm et al., 2022) AI has become a significant topic to investigate. The existing literature has largely focused on the various ways in which AI use can steer creativity and innovation (De Jonge et al., in press) as well as the enablers and inhibitors of AI acceptance in the workplace, with an emphasis on employee individual differences and characteristics as well as broader organizational structures (Yu et al., 2023). More specifically, previous research has shown that gender and culture influence employee perceptions of AI, with females and non-white minorities often exhibiting higher technology anxiety and more negative attitudes towards AI (Rosen and Weil, 1995; Brown et al., 1998; McClure, 2018; see also Schepman and Rodway, 2023). Moreover, self-efficacy, or the belief in one’s ability to use technology, is a significant predictor of AI use (Bandura, 1977; Compeau and Higgins, 1995; Vargas et al., 2018). Finally, introversion (Schepman and Rodway, 2023) as well as compatibility of AI with one’s personal values and experiences increase the likelihood of adopting AI (Chung, 2014).

Besides antecedents related to employee demographics and individual differences, various contextual factors in organizations have been proposed to influence AI attitudes and use. For instance, larger organizations which are more likely to innovate and benefit from technology adoption are more likely to encourage the integration of AI in daily work tasks (Aboelmaged, 2014). Moreover, organizational readiness, in the sense of availability of the required resources for AI integration (Alsheibani et al., 2018) is another very important predictor of successful integration of AI (Pumplun et al., 2019). Yet, another crucial antecedent of AI acceptance is organizational culture: when organizations encourage innovative thinking and creativity, they are more likely to promote the acceptance and integration of AI (Mikalef and Gupta, 2021). Indeed, when creativity is cultivated in an organization, and when employees are encouraged to innovate and think out-of-the-box, employees are more positive towards AI, which they view as a valuable tool that enables them to perform their tasks (Vinchon et al., 2023).

Our study examines the role of organizational climate as a critical factor influencing AI acceptance. Organizational climate captures employees’ perceptions of the workplace, including shared views on organizational events, practices, objectives, and endorsed behaviors (Pullig et al., 2002). We argue that organizational climate is essential in predicting employee AI attitudes because it shapes how employees interpret organizational goals, expected outcomes, and their own actions within the organization (Jones and James, 1979). One specific type of organizational climate that can significantly influence how employees perceive and embrace AI is competitive climate (Nerstad et al., 2013). A *competitive climate* involves elements like comparing individual performance to peers within a work unit, perceiving competition from colleagues, and undergoing frequent evaluations of one’s status (see Ames and Ames, 1984; Nerstad et al., 2013; Černe et al., 2014a,b). In such a climate, rewards typically favor top-performing employees, providing them financial incentives, promotions, recognition, or enhanced status. Considering that AI aligns with the competitive nature of this climate and fosters employee creativity and innovation (Amabile, 2020; De Jonge et al., in press), we propose that a competitive climate positively relates to both favorable attitudes towards AI and its actual use in the workplace.

Moreover, considering that in competitive climates employees are motivated to outperform their peers (Brown et al., 1998), they might realize over time that AI can provide them with tools and insights that give them a competitive advantage. This realization could lead to a more favorable attitude towards AI as a helpful resource for achieving their professional goals over time. In addition, in competitive climates, employees often strive for recognition and rewards (Nerstad et al., 2013; Černe et al., 2014a,b). If AI facilitates the attainment of desired rewards and recognition by employees, employees might come to view AI as a valuable ally. Over time, as they experience the benefits of AI-assisted work, their acceptance of AI may increase. Accordingly, we hypothesize that the positive effect of competitive climate on AI attitudes and AI use will become stronger over time. Based on the above, we stated the following hypothesis:

*Hypothesis 1a*: Competitive climate will be positively related to employees’ favorable attitudes towards AI over time.

*Hypothesis 1b*: Competitive climate will be positively related to employees’ actual use of AI over time.

### The moderating role of leader’s power construal as responsibility or opportunity

While organizational climate offers valuable insights into the general work environment, it is important to recognize that predicting employee AI acceptance requires considering various other essential factors. For instance, within competitive climates, there is a strong dependency on leaders because the distribution of scarce resources, such as salary, promotions, and rewards, relies on leaders’ decisions (*cf.* Wayne and Ferris, 1990). Therefore, how leaders interact with their employees in competitive organizational climates becomes a critical factor in anticipating employee outcomes (see also Fousiani and Wisse, 2022). Additionally, employees often perceive competition at work as potentially detrimental and threatening, increasing the likelihood of self-exploitation (Kohn, 1992). More specifically, in competitive organizational climates, employees may put in substantial effort yet still not achieve the expected organizational rewards, including salary, promotions, rewards, and status (Clark et al., 2016). The possibility of incurring losses despite significant time and effort investment can lead to feelings of uncertainty and stress among employees (Fletcher et al., 2008). In fact, within competitive climates, where resources are scarce, employees might perceive AI as an additional threat (see Schepman and Rodway, 2023) fearing that it could outperform their skills and potentially replace them, with their efforts going unnoticed (Wilkens, 2020; Schepman and Rodway, 2023; Yu et al., 2023). The vulnerability felt by employees in such climates could be therefore a factor contributing to their reluctance to adopt and utilize AI.

The challenges posed by competitive organizational climates emphasize the degree to which employees rely on their leaders and the strategies these leaders employ for their survival and success in organizations (Fousiani and Wisse, 2022). Accordingly, in this study, we introduce leadership as an important moderator in the relationship between competitive climate and AI acceptance. Leaders can utilize the inherent power that accompanies their role in a pro-social manner, instilling a sense of security among employees in competitive climates and valuing their contributions, or employ it in a more self-serving way, intensifying employees’ feelings of threat and insecurity (Wisse and Rus, 2012) and possibly their concern about being replaceable (Raelin, 2003). In this study, we propose that the way a leader construes their power, either as a *responsibility* to support and empower their employees in achieving their goals or as an *opportunity* to advance their own personal self-interests, influences the relationship between a competitive work climate and employee attitudes towards and use of AI.

Leaders who perceive their power as responsibility demonstrate a genuine interest in their employees’ outcomes. They actively seek input and advice from their employees and take their concerns into consideration before making decisions on their behalf. Moreover, leaders who construe their power as responsibility employ their power not only for their own benefit but also to meet the needs and desires of their employees (Sassenberg et al., 2014; De Wit et al., 2017; Scholl et al., 2017, 2018; Fousiani and Wisse, 2022). In contrast, leaders who view their power as opportunity to advance their own goals, perceive it as granting them the freedom to make independent decisions, often without consulting their employees or taking their viewpoint into account, primarily driven by their own interests (Sassenberg et al., 2014; De Wit et al., 2017; Scholl et al., 2017, 2018; Fousiani and Wisse, 2022). Building on the above, in this study we argue that competitive climate is positively related to AI acceptance over time only when leaders construe their power as responsibility towards their employees. Under such circumstances, employees are less likely to focus on negative aspects of the competitive climate and see it as a threat and are more prone to recognize the advantages it can bring, notably in encouraging creativity and innovation in their tasks. This perception of competitive climate actually supports the utilization of AI to steer their performance. Conversely, when leaders construe their power as opportunity to serve self-interested goals, competitive climate is associated with unfavorable AI attitudes and decreased AI use over time because in this case, employees focus on the undesirable aspects of competitive climates, such as feeling threatened by continuous evaluations or being dispensable, which will hinder their favorable perception of AI. Consequently, based on the above we formulated the following hypothesis:

The relationship between competitive climate and employees’ favorable attitudes towards AI and AI use over time depends on whether leaders construe their power as responsibility or as opportunity. More specifically:

*Hypothesis 2a*: The relationship between competitive climate and employees’ favorable attitudes towards AI and AI use over time is positive when leaders construe their power as responsibility.

*Hypothesis 2b*: The relationship between competitive climate and employees’ favorable attitudes towards AI and AI use over time is negative when leaders construe their power as opportunity.

### Overview of the present research

To test our hypotheses, we conducted a field study among employees and conducted an experiment. The field study encompassed three waves, each separated by 2 to 3 weeks. At Time 1, we measured participants’ perception of a competitive organizational climate (independent variable), and at Time 2, we assessed leaders’ power construal as responsibility or opportunity as perceived by employees. Furthermore, both at Time 2 and Time 3, we assessed employee attitudes towards AI and their actual use of AI in the workplace. For ecological validity, we adapted the scale assessing the actual use of AI to specifically address a relevant context: utilizing AI to address workplace conflicts or sharp disagreements. To investigate the impact of competitive climate on AI-related outcomes over time, we controlled for these variables at the preceding time (e.g., the dependent variables were attitudes towards AI and AI use at Time 3, controlling for the same variables at Time 2). At Time 2, participants were initially presented with items assessing power construal, followed by the items assessing attitudes and actual use of AI in the workplace.

In our experiment, we utilized a 2 (competitive climate: high vs. low) by 2 (power construal of the leader: high responsibility vs. high opportunity) design, similar to Fousiani and Wisse (2022). Participants were tasked with immersing themselves in the role of an employee within the provided organizational context, which included information about the competitive climate and the leader’s power construal. Subsequently, we evaluated participants’ attitudes towards AI within that specific organizational setting. To extend Study 1 and augment the experiment’s realism, subsequent to the assessment of participants’ AI attitudes, we presented participants with a concise scenario depicting a disagreement/dispute between themselves (in the employee role) and their leader. We then solicited participants’ views on employing AI to resolve the depicted disagreement and their intention to use AI to resolve the disagreement/dispute. Participation was voluntary and confidential and we secured approval from the university’s ethics committee for both studies before commencing data collection.

## Study 1

### Method

#### Study design and participants

A total of 316 participants from the United Kingdom (UK) were recruited (53% female; *M_age_* = 38.28, SD = 10.61, ranging from 22 to 65 years old) for the 1st wave of the study. Participants were recruited through Prolific and were employees working in various organizations in the UK. We exclusively recruited fulltime employees (*M_hours/week_* = 38.80, SD = 3.80). These employees held supervised positions, meaning they had a supervisor. Participants’ prior work experience was *M_years_* = 5.50, SD = 5.26. Participants holding a managerial/supervisory role in their organization were excluded from the study and could not take part. 290 completed the 2nd and 237 partook the 3rd wave of our study resulting in 92 and 75% response rate, respectively. Participants additionally provided information about their highest level of education attained with 35% being high school graduates, 45% holding a Bachelor’s degree, 18% holding a Master’s degree and the remaining 2% holding a post-graduate title. Each survey took approximately 15 min to complete, and participants were compensated about £3.00 per completed survey (for all three waves). To ensure that the study has sufficient statistical power to detect the hypothesized effects with a reasonable level of confidence, we conducted a sensitivity power analysis with GPower 3.1. The analysis was conducted, indicating 95% power to identify a small to medium effect size of *f^2^* = 0.06.

#### Procedure

We assessed participants’ demographics and competitive climate at Time1. Power construal of the leader as responsibility or as opportunity was assessed at Time 2. Finally, participants’ attitudes towards AI and actual use of AI were measured both at Time 2 and Time 3. Upon completing the study’s 3rd wave, participants were thanked, debriefed, and compensated for their participation.

#### Measures

##### Competitive climate

Similar to previous studies (e.g., Wisse et al., 2019; Fousiani and Wisse, 2022) we used the performance climate subscale of the motivational climate scale (Nerstad et al., 2013) to measure employees’ perceptions of competitive climate. Participants rated the extent to which they experience climate within their workplace as competitive using the eight-items of this scale. A sample item included “In my department/work group, it is important to achieve better than others.” Items were measured on a Likert scale ranging from 1 = *strongly disagree* to 7 = *strongly agree*. Cronbach’s alpha was high at *α* = 0.91.

##### Power construal

Participants were requested to rate the extent to which they experienced their leaders’ construal of power as opportunity or responsibility with the 6-item scale of De Wit et al. (2017). Three items assessed each construct of power construal. A sample item for power as responsibility was “In my work, my supervisor tends to see his/her power in terms of the responsibility to ensure that important goals of his/her subordinates are met.” A sample item for power as opportunity was “In my work, my supervisor tends to see his/her power in terms of the opportunity that it gives him/her to tell subordinates what to do without having to ask them what they actually want to do” (1 = *not at all true* to 7 = *absolutely true*). Cronbach’s alpha was α = 0.82 for power as responsibility and α = 0.87 for power as opportunity.

##### Favorable attitudes towards AI

We assessed participants’ attitudes towards AI using the 20-item scale of Schepman and Rodway (2020). A sample item measuring unfavorable attitudes was: “I think Artificial Intelligence is dangerous,” while a sample item designed to evaluate favorable attitudes towards AI was: “Artificial Intelligence is exciting,” (1 = *strongly disagree,* 7 = *strongly agree*). Cronbach’s *α* = 0.76. It is important to note that we employed a reverse coding method for the unfavorable items, allowing us to get a score indicating a participant’s overall attitude towards AI using all 20 items.

##### Actual use of AI in the workplace

Finally, we inquired about the frequency of AI use that our participants perform in their workplace, using a 4-item scale, adapted from Draxler et al. (2023), e.g., “I use Artificial Intelligence to find new perspectives to resolve conflict in the workplace” with their answers spanning between 1 = *very rarely/almost never* to 7 = *very often*. Cronbach’s *α* = 0.97.

##### Control variables

We controlled for sector effects (seven categories) given that industries vary in their culture and norms. Not accounting for said effects would have made it more challenging to discern whether the observed attitudes are truly linked to the competitive climate or are influenced by industry. Further, we controlled for age (measured in years) to make this factor’s effects on AI use easier to isolate given the varying degree of familiarity with technology that different age groups are shown to have in the literature (Venkatesh et al., 2003).

**Figure 2:**
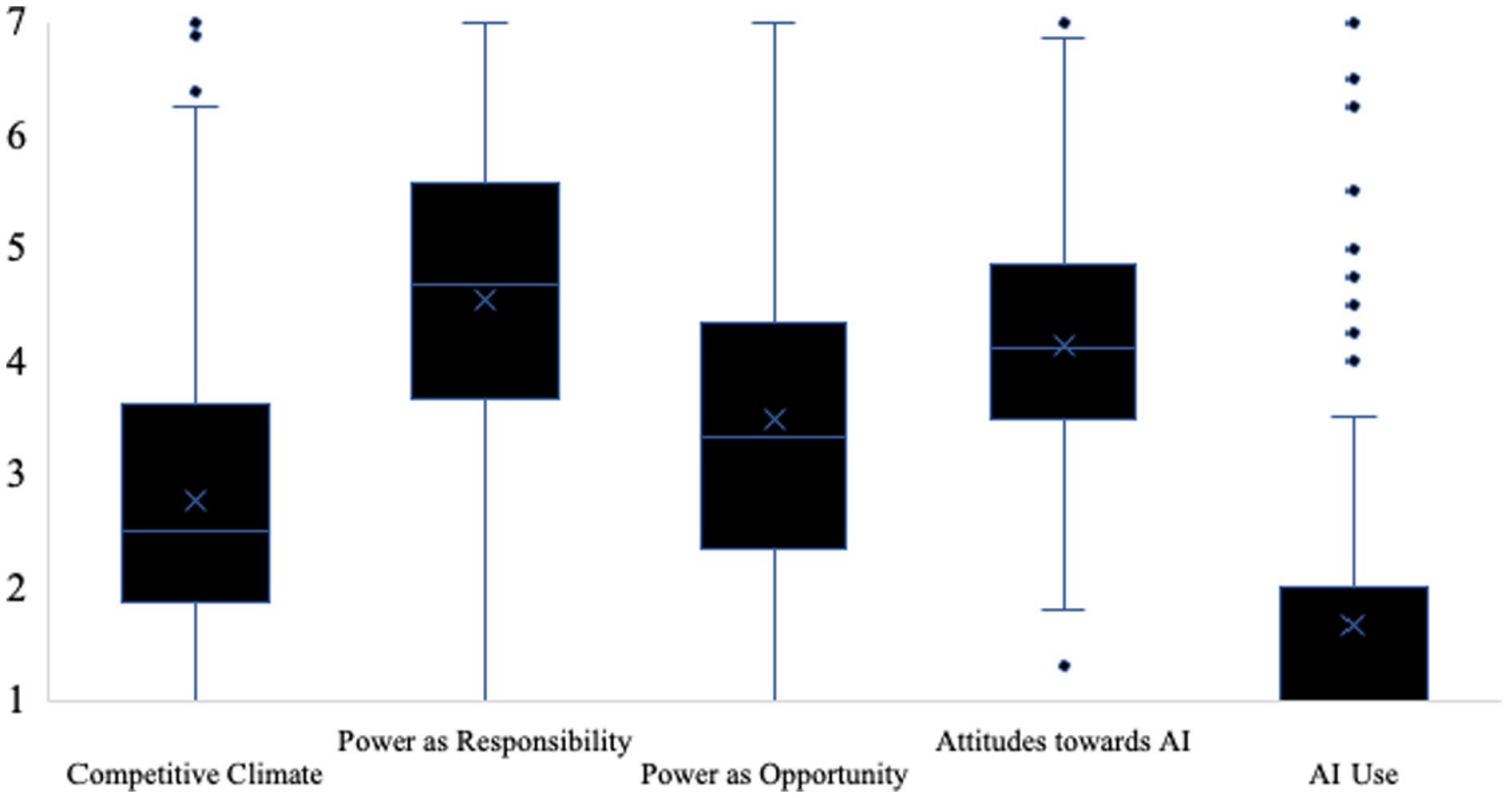
Box plot illustrating variability of participants’ responses across scales (study 1). Competitive climate was measured on a 7-point scale (1 = strongly disagree to 7 = strongly agree). Leader power as responsibility and as opportunity were also measured on a 7-point scale 1 = not at all true to 7 = absolutely true. Attitudes towards AI’s responses ranged from 1 = strongly disagree and 7 = strongly agree. Answers for AI use spanned between 1 = very rarely/almost never to 7 = very often.

For a representation of the variability in participants’ responses see Figure 2. For a complete list of items see online supplementary material.

### Results

Correlations between focal variables, means and standard deviations are presented in Table 1. We first conducted a Confirmatory Factor Analysis with MPlus 8 (Muthén and Muthén, 2017) to ensure that our variables were distinct from one another. In the analysis, we included competitive climate, power construal, employees’ favorable attitudes towards AI and actual use of AI. The model fit was acceptable (*χ^2^* = 1447.448, *df* = 653, *p* < 0.001; RMSEA = 0.06 [CI 0.057; 0.066]; CFI = 0.89; TLI = 0.88; SRMR = 0.067).^1^

**Table 1:** Pearson correlation coefficients, means, and standard deviations (study 1).

	1	2	3	4	5	6	7	M (SD)
Competitive climate^a^	1	−0.00	0.34^**^	0.10	0.27^**^	−0.00	−0.19^**^	2.77 (1.28)
Leader power as responsibility^b^		1	−0.28^**^	0.13^*^	−0.02	−0.05	−0.04	4.54 (1.25)
Leader power as opportunity^b^			1	−0.12	0.16^*^	0.05	−0.01	3.48 (1.50)
Attitudes towards AI^c^				1	0.20^**^	0.01	−0.09	4.15 (1.11)
AI use^d^					1	−0.16^*^	−0.15^*^	1.67 (1.24)
Age (in years)						1	−0.02	38.28 (10.61)
Sector^e^							1	5.43 (1.88)

a Competitive climate was measured on a 7-point scale (1 = strongly disagree to 7 = strongly agree).

b Leader power as responsibility and as opportunity were also measured on a 7-point scale 1 = not at all true to 7 = absolutely true.

c Attitudes towards AI’s responses ranged from 1 = strongly disagree and 7 = strongly agree.

d Answers for AI use spanned between 1 = very rarely/almost never to 7 = very often.

e Sector comprised seven different categories. ^*^p < 0.05; ^**^p < 0.01.

### Hypothesis testing

#### Effects of competitive climate and leader power construal on employee favorable attitudes towards AI over time

**Figure 3:**
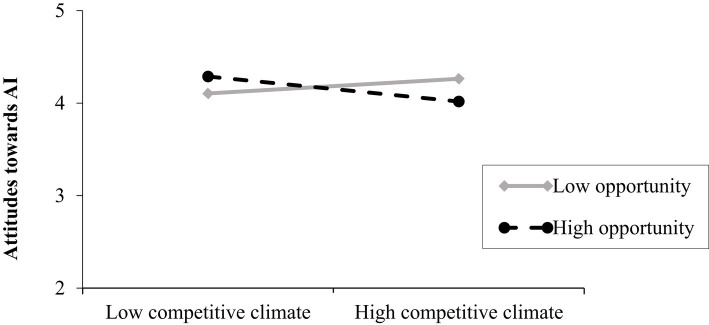
Relationship between competitive climate and favorable attitudes towards AI over time as a function of leader’s power construal (study 1). Competitive climate was measured on 7-point scale (1 = strongly disagree to 7 = strongly agree). Leader power construal as opportunity was measured on a 7-point scale (1 = not at all true, to 7 = absolutely true). Favorable attitudes towards AI was also measured on a 7-point scale (1 = strongly disagree, 7 = strongly agree).

The overall model was significant *R*^2^ = 0.87, *F*(8, 228) =190.37, *p* < 0.001. Opposite to Hypothesis 1a, the main effect of competitive climate on attitudes towards AI at T3 (controlling for attitudes towards AI at T2) was not significant. Moreover, neither leader power as responsibility nor leader power as opportunity significantly influenced employees’ attitudes towards AI over time. The competitive climate by leader power as responsibility interaction on employees’ favorable attitudes towards AI over time was also not significant Δ*R*^2^ = 0.002, *F*(1,228) = 2.87, *p* = 0.09. These results do not provide support for Hypothesis 2a. However, the interaction between competitive climate and leader power as opportunity on attitudes towards AI at T3 (controlling for attitudes towards AI at T2) came out significant Δ*R*^2^ = 0.003, *F*(1, 228) = 6.00, *p* = 0.015 and showed a negative relationship between competitive climate and employees’ favorable attitudes towards AI when leaders’ power construal as opportunity is high (*b* = −0.10, *SE* = 0.04, *p* = 0.005; 95% CI [−0.18; −0.03]). The effect of competitive climate on employees’ favorable attitudes towards AI over time was not significant at low levels of power as opportunity (see Figure 3). These results provide support for Hypothesis 2b. For the relevant statistics see Table 2.

**Table 2:** Relationship between competitive climate, attitudes towards AI, and actual use of AI over time as a function of leader’s power construal (study 1).

	Favorable attitudes towards AI at T3^c^ (controlling for attitudes towards AI at T2^c^)				Actual AI use at T3^d^ (controlling for AI use at T2^d^)			
Predictor	*B*	*t*	*p*	95% *CI*	*B*	*t*	*p*	95% *CI*
Constant	0.21 (0.17)	1.26	0.21	−0.12; 0.55	1.07 (0.33)	3.28	<0.01	0.43; 1.71
Competitive climate^a^	−0.02 (0.02)	−0.90	0.37	−0.07; 0.03	0.14 (0.05)	2.57	0.01	0.03; 0.24
Power as responsibility^b^	0.02 (0.02)	0.71	0.48	−0.03; 0.06	0.02 (0.05)	0.35	0.73	−0.08; 0.12
Competitive climate^a^ × Power as responsibility^b^	−0.03 (0.02)	−1.70	0.09	−0.06; 0.00	−0.02 (0.04)	−0.49	0.63	−0.09; 0.05
Power as opportunity^b^	−0.02 (0.02)	−1.17	0.24	−0.06; 0.02	0.06 (0.05)	1.33	0.19	−0.03; 0.15
Competitive climate^a^ × Power as opportunity^b^	−0.03 (0.01)	−2.45	0.02	−0.06; −0.01	−0.07 (0.03)	−2.30	0.02	−0.13; −0.01
Attitudes towards AI at T2^c^	0.97 (0.03)	37.57	<0.01	0.91; 1.02	–	–	–	–
AI use at T2^d^	–	–	–	–	0.60 (0.05)	12.10	<0.01	0.50; 0.69
Age (in years)	0.00 (0.00)	0.51	0.61	−0.00; 0.01	−0.01 (0.01)	−0.86	0.39	−0.02; 0.01
Sector^e^	−0.02 (0.01)	−1.36	0.18	−0.05; 0.01	−0.03 (0.03)	−0.84	0.40	−0.09; 0.04

a Competitive climate was measured on a 7-point scale (1 = strongly disagree to 7 = strongly agree).

b Leader power as responsibility and as opportunity were also measured on a 7-point scale 1 = not at all true to 7 = absolutely true.

c Favorable attitudes towards AI at T2 and T3 were measured on a 7-point scale (1 = strongly disagree, 7 = strongly agree).

d Answers for AI use at T2 and T3 spanned between 1 = very rarely/almost never to 7 = very often.

e Sector comprised seven different categories.

#### Effects of competitive climate and leader power construal on employee actual use of AI over time

**Figure 4:**
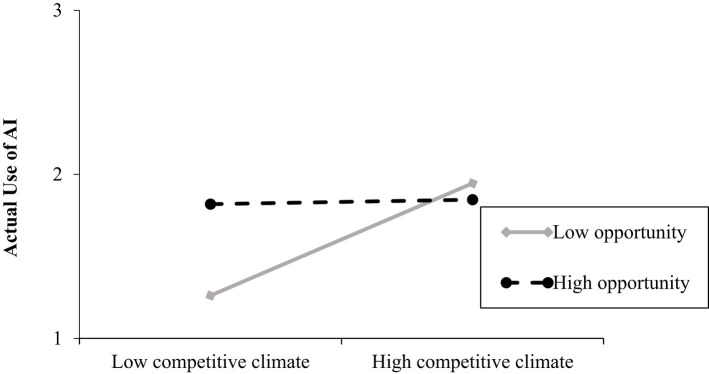
Relationship between competitive climate and actual use of AI over time as a function of leader’s power construal (study 1). Leader power construal as opportunity was measured on a 7-point scale (1 = not at all true, to 7 = absolutely true). Competitive climate was measured on 7-point scale (1 = strongly disagree to 7 = strongly agree). AI actual use was also measured on a 7-point scale (1 = very rarely/almost never to 7 = very often).

The overall model was significant *R*^2^ = 0.48, *F*(8, 228) =25.82, *p* < 0.001. Consistent to Hypothesis 1b, the main effect of competitive climate on actual use of AI at T3 (controlling for use of AI at T2) was positive and significant (*b* = 0.14, *SE* = 0.05, *p* = 0.01; 95% CI [0.03; 0.24]). Neither leader power as responsibility nor leader power as opportunity significantly influenced employees’ actual use of AI over time. Moreover, opposite to Hypothesis 2a, the competitive climate by leader power as responsibility interaction on employees’ actual use of AI over time was not significant. However, consistent to Hypothesis 2b, the interaction between competitive climate and leader power as opportunity on actual use of AI at T3 (controlling for use of AI at T2) came out significant Δ*R*^2^ = 0.01, *F*(1, 228) = 5.29, *p* = 0.02 and showed a positive relationship between competitive climate and employees’ actual use of AI when leaders’ power construal as opportunity is low (*b* = 0.22, *SE* = 0.08, *p* = 0.005; 95% CI [0.07; 0.38]). The effect of competitive climate on employees’ actual use of AI over time was not significant at high levels of power as opportunity (see Figure 4). These results provide support for Hypothesis 2b. For the relevant statistics see Table 2.

### Discussion

Study 1 was a field study investigating the relationship between competitive climate on the one hand, and employees’ attitudes towards AI and AI use over time on the other hand. Furthermore, Study 1 investigated the moderating effect of power construal of the leader as responsibility or as opportunity in this relationship. The effect of competitive climate at Time 1 on *AI attitudes* at Time 3 (controlling for AI attitudes at Time 2) was not significant. However, the effect of competitive climate at Time 1 on *AI use* at Time 3 (controlling for AI use at Time 2) was significant and positive. The results did not provide support for Hypothesis 1a, but they did provide support for Hypothesis 1b. Opposite to Hypothesis 2a, competitive climate did not interact with leader’s power as responsibility in the prediction of employee AI attitudes or use of AI. In line with our expectations, competitive climate at Time 1 interacted with leader’s power as opportunity at Time 2 in the prediction of both (a) *attitudes towards AI* at Time 3 (controlling for AI attitudes at Time 2) and (b) *actual use of AI* at Time 3 (controlling for AI use at Time 2). More specifically, results showed that competitive climate is negatively related to employees’ favorable attitudes towards AI and to their actual use of AI over time when the leader construes their power as opportunity. These results provide full support for Hypothesis 2b.

In conclusion, Study 1 provided valuable insights into the dynamics of competitive climate and its influence on AI acceptance. The results also illuminated the moderating role of leader power as opportunity in the relationship between competitive climate and employee attitudes and use of AI over time. However, the study did not yield a significant interaction between competitive climate and leader’s power as responsibility, suggesting that competitive climates may positively influence AI acceptance, particularly in terms of actual AI use, regardless of leaders’ prosocial power use (as responsibility). In contrast, when leaders exhibit opportunistic behaviors by using their power as opportunity, competitive climates are associated with decreased AI acceptance. Nevertheless, it is worth noting that the non-significant interaction between competitive climate and leader’s power as responsibility may be due to the limitations of the measure used to assess power as responsibility, which consisted of only three items and is a relatively recent construct (de Wit et al., 2017; see also Fousiani and Wisse, 2022 regarding the limitations of the power construal measure).

In order to strengthen our theoretical framework and enhance the study’s internal validity, we conducted Study 2, an experiment in which we manipulated competitive climate and leader power construal within vignettes. This approach allowed us to investigate employee attitudes towards AI in a controlled environment, thereby mitigating limitations commonly encountered in field studies. Additionally, we increased the study’s realism by presenting participants with a specific scenario in which they imagined an event involving themselves (in the employee role) and their leader, and then assessed their attitudes towards AI for resolving the scenario and their intentions to use AI for a solution.

## Study 2

### Method

#### Participants

A total of 150 participants took part in this experiment,^2^ (56.7% female; *M*_age_ = 38.16, SD = 10.98, ranging between the age of 23 and 67). Participants were recruited through Prolific. Similar to Study 1, all participants were fulltime employees (*M_work/h_* = 38.53; SD = 3.55) working for various organizations in the UK. Participants had previous work experience of *M_years_* = 10.31, SD = 9.16. Participants holding a managerial/supervisory role in their organization were not eligible for the study and were not allowed to take part. One participant was a primary school graduate, 25.3% had finished high school, 46% held a Bachelor’s and 24.7% a Master’s degree, while 3.3% were PhD graduates. A sensitivity power analysis yielded an 80% chance of detecting a medium to large effect size of *f* = 0.34.

#### Experimental design and procedure

Participants were randomly assigned to one of four conditions based on a 2 (competitive climate: high vs. low) x 2 (leader’s power construal: high opportunity vs. high responsibility) between-subjects design. We presented the participants with vignettes featuring descriptions of their presumed supervisor, Bill, who construed their power as either responsibility or opportunity similar to Fousiani and Wisse (2022). More specifically, participants in the high power as opportunity condition read:

“Your supervisor, Bill, is a person who sees his power as a great opportunity to influence others to his own advantage and as a chance to tell others what to do. He is the type of leader who feels that he can focus on the opportunities to achieve goals that he finds important for himself. For instance, in a recent conversation with him, Bill told you that he is using the possibility that his position as a supervisor gives him to make decisions that determine his own outcomes as well as those of his subordinates (ranging from the tasks to be performed, to the trainings to attend, and the bonus one is eligible for). Bill indeed always makes use of this opportunity. His motto is: Power gives you the chance to look out for your own interest and you should always use that option.” In the condition high power as responsibility, participants read “Your supervisor, Bill, is a person who sees his power as a great responsibility towards others and as an obligation towards other people to take care of things that need to be done. He is the type of leader who feels responsible for ensuring that important group goals are met. For instance, in a recent conversation with him, Bill told you that he is well aware of the responsibility that his position as a supervisor gives him to make decisions that have important consequences for himself but also for his subordinates (ranging from the tasks to be performed, to the trainings to attend, and the bonus one is eligible for). Bill indeed always takes care of these commitments. His motto is: Power gives you the duty to look out for other people’s interest and you should always do that.”

Additionally, participants were exposed to one of the two work climate descriptions, depending on the condition they were assigned to, namely highly competitive or low competitive. Manipulation of competitive climate was identical to Wisse et al. (2019) and Fousiani and Wisse (2022) (see Supplementary material for the complete vignettes). Manipulation checks for power construal and competitive climate followed the vignettes.

After giving adequate time so that the participants immerse themselves into the experimental conditions, we asked them to fill in a scale measuring their attitudes towards AI in that specific work context. Afterwards, we presented participants with an imaginary conflict between themselves and their supervisor. The conflict was about how the participant (employee) could spread their working hours across the week. More specifically, participants read: “*In the company you are working at, a standard working week is 36 h. Your supervisor, Bill, wants you to work fewer hours per day but more days per week. You disagree. You want to work more hours per day but fewer days per week. How will you approach this disagreement/conflict with your supervisor, Bill*? This vignette was previously used by Fousiani et al. (2022). Following the vignette, we asked participants to indicate their attitudes towards AI for the resolution of the conflict at hand and the likelihood (intention) of using AI to resolve this conflict. Subsequent to the filling in of survey questions pertaining to demographic information, the participants were debriefed and thanked for participating. Compensation of £1.20 for their approximately 10-min involvement promptly ensued upon experimental completion.

#### Measures

##### Manipulation checks

Two items served as manipulation checks for power construal (power as opportunity: “Bill uses the power that comes with his supervisory position as an opportunity to influence his subordinates to his own advantage”; power as responsibility: “Bill uses the power that comes with his supervisory position as a means to fulfill his responsibility towards his subordinates”; 1 = *not at all*, 7 = *to a great extent*). Manipulation checks for competitive climate were assessed with three items (e.g., “The climate in this company is competitive”; 1 = *strongly disagree*, 7 = *strongly agree*) similar to Fousiani and Wisse (2022).

##### Favorable attitudes towards AI (general)

Participants’ favorable attitudes towards using AI in the work context described in the scenario was assessed using two adapted items from Draxler et al. (2023) (e.g., “How likely is AI to be helpful in your organization, as described in the scenario you read?”; 1 = *very unlikely,* 7 = *very likely*). Cronbach’s *α* = 0.82.

##### Favorable attitudes towards AI for the resolution of an issue (specific)

Participants’ attitude towards AI for managing a specific issue (dispute with leader) was assessed with an adapted 2-item scale (Draxler et al., 2023), e.g., “AI can be a positive force in managing this conflict with my supervisor, Bill.” (1 = *strongly disagree,* 7 = *strongly agree*). Cronbach’s *α* = 0.97.

##### Likelihood of using AI to resolve an issue

To assess a participant’s intention to use AI to resolve a specific issue (dispute with leader), a 2-item adapted scale was employed (Draxler et al., 2023), e.g., “I would use AI to find creative solutions to this disagreement/conflict with my supervisor, Bill.” (1 = *very unlikely,* 7 = *very likely*). Cronbach’s *α* = 0.96.

##### Control variables

Similar to study 1, age was used as a control variable. Additionally, we controlled for participants’ weekly working hours.

**Figure 5:**
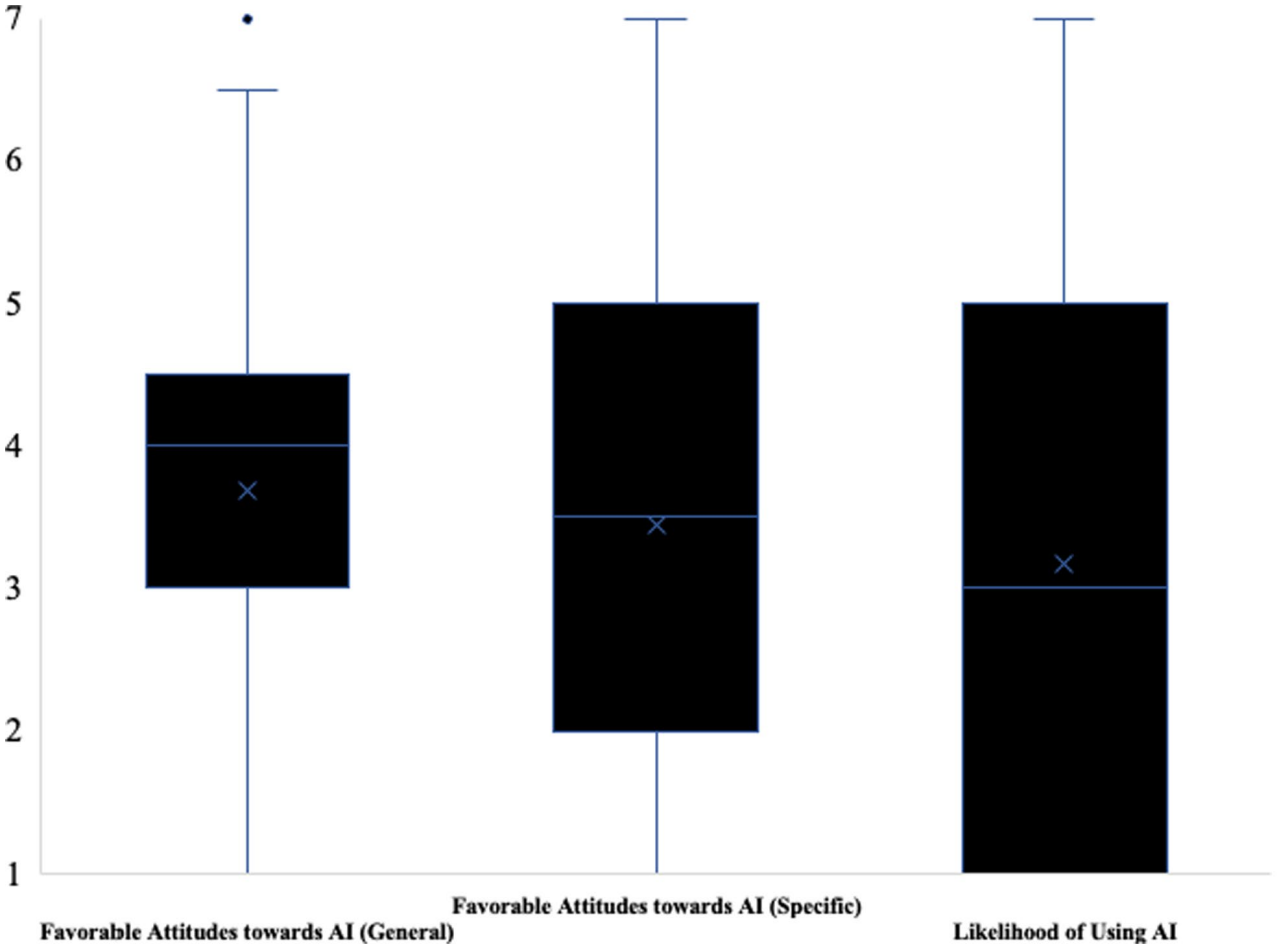
Box plot illustrating variability of participants’ responses across scales (study 2). Favorable attitudes towards AI (general) were measured as (1 = very unlikely, 7 = very likely), favorable attitudes towards AI (specific) were measured as (1 = strongly disagree, 7 = strongly agree). The likelihood of using AI was measured as (1 = very unlikely, 7 = very likely).

For a representation of the variability in participants’ responses see Figure 5. For a complete list of vignettes and items see Supplementary material.

### Results

Favorable attitudes towards AI (*M* = 3.68, SD = 1.36) was positively related to favorable attitudes towards AI for the resolution of the issue at hand (a conflict) (*M* = 3.44, SD = 1.74) (*r* = 0.33, *p* < 0.001) and positively related to the likelihood of using AI in order to resolve an issue at hand (*M* = 3.17, SD = 1.83) (*r* = 0.33, *p* < 0.001). Moreover, favorable attitudes towards AI for the resolution of an issue was positively related to AI use (*r* = 0.84, *p* < 0.001). Our control variables, namely age and working hours per week, did not significantly correlate with any other variables.

### Manipulation checks

To determine whether our manipulations worked as intended, we ran a multivariate analysis of variance with competitive climate and power construal as fixed factors and manipulation check items for competitive climate, power as opportunity, and power as responsibility as dependent variables. The main effect of competitive climate on the competitive climate manipulation check items was significant *F*(1, 146) = 14.08, *p* < 0.001, *η*2 = 0.09 and showed that participants in the high-competitive climate condition indicated having experienced the work climate as more competitive (*M* = 6.69, SD = 0.56) as opposed to participants in the low-competitive climate condition (*M* = 1.52, SD = 0.90). The main effect of power construal (opportunity vs. responsibility) on the perception of leader’s construal of power as opportunity was also significant *F*(1, 146) = 198.47, *p* < 0.001, *η*2 = 0.58 and showed that participants in the high opportunity condition (*M* = 6.39, SD = 1.13) as opposed to participants in the high responsibility condition (*M* = 3.04, SD = 1.91). Similarly, the power construal condition had a significant effect on participants’ experience of the leader’s construal of power as responsibility *F*(1, 146) = 108.74, *p* < 0.001, *η*2 = 0.43. Participants in the high power as responsibility condition indicated having experienced their leader’s power as responsibility (*M* = 6.13, SD = 0.92) to a greater extent as compared to participants in the high power as opportunity condition (*M* = 3.56, SD = 1.91). We can conclude that our manipulations worked as intended.

### Hypothesis testing

#### Effects of competitive climate and leader power construal on employee favorable attitudes towards AI (general)

Competitive climate was coded as: 1 = low, 2 is high. Similarly, leader power as responsibility and leader power as opportunity were coded as 1 = high opportunity and 2 = high responsibility.

**Figure 6:**
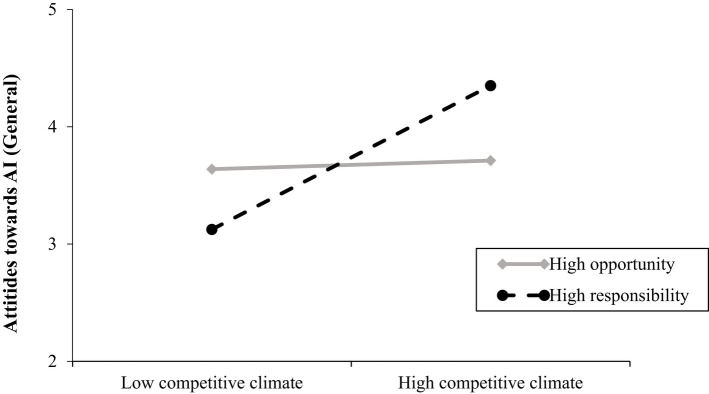
Relationship between competitive climate and favorable attitudes towards AI (general) as a function of leader’s power construal (study 2). Competitive climate was coded as: 1 = low, 2 is high. Leader’s power construal was coded as 1 = high opportunity and 2 = high responsibility. Favorable attitudes towards AI (general) were measured on a 7-point scale (1 = very unlikely, 7 = very likely).

The overall model was significant *R*^2^ = 0.09, *F*(5, 131) =2.50, *p* = 0.03. Contrary to Hypothesis 1a, the main effect of competitive climate on favorable attitudes towards AI was not significant. Power construal did have a significant and negative effect on favorable attitudes towards AI showing that participants have more favorable attitudes towards AI when leaders construe power as opportunity rather than responsibility. The competitive climate by leader power construal interaction on favorable attitudes towards AI was significant Δ*R*^2^ = 0.04, *F*(1, 131) = 5.24, *p* = 0.02 and showed that participants have more favorable attitudes towards AI when climate is competitive and when leaders construe power as responsibility (*b* = 1.23, *SE* = 0.35, *p* = 0.007; 95% CI [0.53; 1.92]) (see Figure 6). The interaction was not significant for the power as opportunity slope (*b* = 0.07, *SE* = 0.35, *p* = 0.84; 95% CI [−0.63; 0.78]). These provide support for Hypotheses 2a but not for Hypothesis 2b. For the relevant statistics see Table 3.

**Table 3:** Relationship between competitive climate, attitudes towards AI and likelihood to use AI as a function of leader’s power construal (study 2).

	Favorable attitudes towards AI (general)^c^				Favorable attitudes towards AI (specific)^d^				Likelihood of using AI^e^			
Predictor	*B*	*t*	*p*	95% *CI*	*B*	*t*	*p*	95% *CI*	*B*	*t*	*p*	95% *CI*
Constant	5.36 (1.89)	2.84	0.01	1.63; 9.09	6.74 (2.16)	3.12	0.00	2.46; 11.01	7.49 (2.37)	3.16	0.00	2.80; 12.17
Competitive climate^a^	−1.08 (0.80)	−1.36	0.18	−2.66; 0.50	−2.46 (0.91)	−2.69	0.01	−4.27; −0.65	−2.04 (1.00)	−2.03	0.04	−4.02; −0.06
Power construal^b^	−1.67 (0.79)	−2.11	0.04	−3.23; −0.10	−2.86 (0.91)	−3.16	0.00	−4.66; −1.07	−2.90 (0.99)	−2.92	0.00	−4.86; −0.94
Competitive climate^a^ x Power construal^b^	1.15 (0.50)	2.29	0.02	0.16; 2.15	1.81 (0.58)	3.14	0.00	0.67; 2.96	1.54 (0.63)	2.43	0.02	0.28; 2.79
Age (in years)	0.00 (0.01)	0.38	0.70	−0.02; 0.03	−0.01 (0.01)	−0.44	0.66	−0.03; 0.02	−0.01 (0.01)	−0.69	0.49	−0.04; 0.02
Working hours per week	−0.01 (0.04)	−0.22	0.83	−0.08; 0.06	0.02 (0.04)	0.44	0.66	−0.06; 0.10	−0.00 (0.04)	−0.02	0.99	−0.09; 0.09

a Competitive climate was coded as: 1 = low, 2 = high.

b Leader power construal was coded as 1 = high opportunity, 2 = high responsibility.

c Favorable attitudes towards AI (general) were measured as (1 = very unlikely, 7 = very likely).

d Favorable attitudes towards AI (specific) were measured as (1 = strongly disagree, 7 = strongly agree).

e The likelihood of using AI was measured as (1 = very unlikely, 7 = very likely).

#### Effects of competitive climate and leader power construal on employee favorable attitudes towards AI for the resolution of an issue (specific)

**Figure 7:**
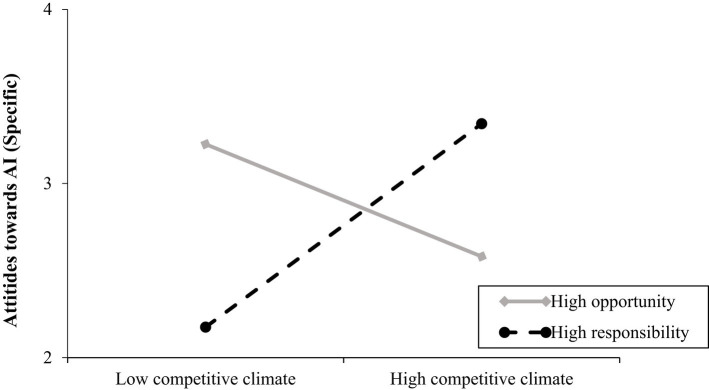
Relationship between competitive climate and favorable attitudes towards AI (specific) as a function of leader’s power construal (study 2). Competitive climate was coded as: 1 = low, 2 is high. Leader’s power construal was coded as 1 = high opportunity and 2 = high responsibility. Favorable attitudes towards AI (specific) were measured on a 7-point scale (1 = strongly disagree, 7 = strongly agree).

The overall model was significant *R*^2^ = 0.09, *F*(5, 131) =2.43, *p* = 0.04. Opposite to Hypothesis 1a, the main effect of competitive climate on favorable attitudes towards AI was significant and negative. Power construal did have a significant and negative effect on favorable attitudes towards AI showing that participants have more favorable attitudes towards AI when leaders construe power as opportunity rather than responsibility. The competitive climate by leader power construal interaction on favorable attitudes towards AI was significant Δ*R*^2^ = 0.07, *F*(1, 131) = 9.87, *p* = 0.002 and showed that participants have more favorable attitudes towards AI for the resolution of an issue (conflict with the leader) when climate is competitive and when leaders construe power as responsibility (*b* = 1.17, *SE* = 0.41, *p* = 0.005; 95% CI [0.37; 1.97]). The interaction was not significant for the power as opportunity slope (*b* = −0.65, *SE* = 0.41, *p* = 0.12; 95% CI [−1.45; 0.16]) (see Figure 7). These provide support for Hypothesis 2a but not for Hypothesis 2b. For the relevant statistics see Table 3.

#### Effects of competitive climate and leader power construal on employee likelihood of using AI for the resolution of an issue

**Figure 8:**
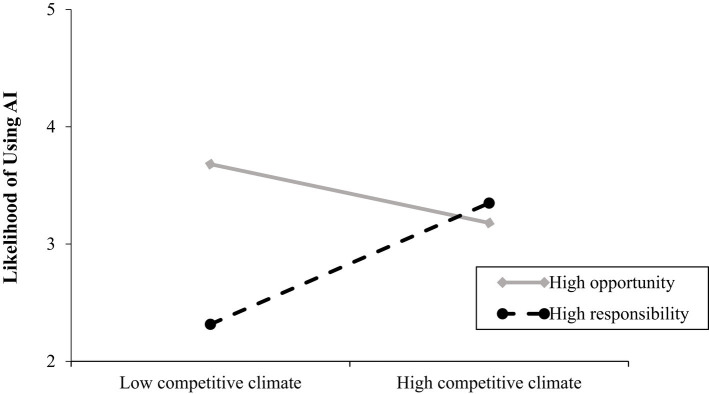
Relationship between competitive climate and likelihood of using AI for the resolution of an issue as a function of leader’ power construal (study 2). Competitive climate was coded as: 1 = low, 2 is high. Leader’s power construal was coded as 1 = high opportunity and 2 = high responsibility. Likelihood of using AI to resolve an issue was measured on a 7-point scale (1 = very unlikely, 7 = very likely).

The overall model was significant *R*^2^ = 0.08, *F*(5, 131) =2.29, *p* < 0.05. Opposite to Hypothesis 1a, the main effect of competitive climate on favorable attitudes towards AI was significant and negative. Power construal did have a significant and negative effect on likelihood of using AI showing that participants have a stronger intention to use AI when leaders construe power as opportunity rather than responsibility. The competitive climate by leader power construal interaction on likelihood of using AI was significant Δ*R*^2^ = 0.04, *F*(1, 131) = 5.88, *p* = 0.02 and showed that participants have a stronger intention to use AI for the resolution of an issue (conflict with the leader) when climate is competitive and when leaders construe power as responsibility (*b* = 1.03, *SE* = 0.44, *p* = 0.02; 95% CI [0.16; 1.91]). The interaction was not significant for the power as opportunity slope (*b* = −0.50, *SE* = 0.45, *p* = 0.26; 95% CI [−1.38; 0.38]) (see Figure 8). These provide support for Hypothesis 2a but not for Hypothesis 2b. For the relevant statistics see Table 3.

### Discussion

Study 2 involved an experiment where we manipulated competitive climate and leader power construal within vignettes. Unexpectedly, the main effect of competitive climate on attitude towards AI was non-significant. Moreover, when asking participants to address a specific issue (a dispute with their leader) the main effect of competitive climate on participants’ AI attitudes and intention to use AI was significant but negative. These findings contradict Hypotheses 1a and 1b. In line with our expectations, the interaction between competitive climate and power construal was found to be significant and results were consistent with our expectations: In conditions of a highly competitive climate, participants exhibited more favorable attitudes towards AI, especially when their leader construed power as responsibility rather than as opportunity. Similarly, when focusing on the specific event of the leader-employee dispute, participants displayed more favorable attitudes towards AI and a stronger intention to use it possibly because they see it as a problem-solving tool, especially when their leader construed their power as responsibility rather than opportunity. These findings complement Study 1 and provide support for Hypothesis 2a and partially 2b.

These unexpected findings pertaining to Hypothesis 1a and 1b might be attributed to the particular issue being examined, namely a dispute between a leader and an employee about working from home or remotely, and may not be generalized to other work-related tasks affecting employees. For instance, when employees encounter conflicts or strong disagreements with their leaders within a competitive work environment, they may perceive the conflict in a particularly adverse or even threatening manner. This perception may be influenced by the sense of dependency that competitive climates create for employees (*cf.* Wayne and Ferris, 1990), potentially leading to a reduced willingness to invest in innovative technologies and creative approaches facilitated by AI. Future research should further investigate the relationship between competitive climate and AI acceptance. Taken together, these results show that the effect of competitive climate alone on AI acceptance may not be consistent; In contrast, we should consider the effect of competitive climate on employee AI acceptance in conjunction with leader power construal.

## General discussion

This research, comprising a field study (Study 1) and an experiment (Study 2), investigated the relationship between competitive organization climate and employee AI attitudes and use. Study 1, a three-wave field study conducted among employees, showed that competitive climate significantly and positively influences AI use over time, supporting Hypothesis 1b. While Hypothesis 1a, which suggested a positive relationship between competitive climate and favorable AI attitudes over time was not supported, Hypothesis 2b was supported by the significant interaction between competitive climate and leader’s power as opportunity. Specifically, Study 1 showed that competitive climate is negatively related to favorable attitudes towards AI and AI use over time when leaders construe their power as opportunity. Nevertheless, the interaction between competitive climate and power as responsibility was not significant, thus failing to support Hypothesis 2a.

Study 2, an experiment, aimed to replicate these results in a controlled setting. This study, unexpectedly revealed a negative effect of competitive climate on AI acceptance (i.e., attitudes and actual use) when addressing a specific issue (handling a dispute with one’s leader), contradicting Hypotheses 1a and 1b. Nonetheless, the interaction between competitive climate and power construal emerged as expected; competitive climate had a positive effect on employee AI attitudes and intention to use AI when leaders construed power as responsibility rather than as opportunity, providing support for Hypothesis 2 (a and partly b). We conclude that the main effect of competitive climate on AI acceptance is not consistent; however, the interaction between competitive climate and leader’s power construal in the prediction of AI acceptance is a robust phenomenon, as seen in both studies.

### Theoretical and practical implications

The theoretical implications of this research are multifaceted. First and foremost, this study highlights that organizational climate can significantly impact AI use over time, shedding light on the role of the organizational work climate in AI acceptance. These results extend previous research suggesting an important effect of organizational dynamics (e.g., organizational climate, leadership) on AI acceptance (for a review, see Yu et al., 2023). Indeed, while existing studies have extensively examined how employee characteristics and demographics affect the acceptance and use of AI (McClure, 2018; Schepman and Rodway, 2023), the role of organizational factors remains underexplored. This study fills this gap by demonstrating the interactive role between competitive climate and leader’s power construal on AI acceptance and use. Furthermore, this research advances the existing body of literature on competitive climate and leadership by pointing out their interaction in the prediction of employee-related outcomes. More specifically, this research highlights the detrimental impact of construing power as opportunity (Study 1) and the favorable impact of construing power as responsibility (Study 2) among employees in competitive work environments (see also Fousiani and Wisse, 2022). Accordingly, these results provide insights that expand the literature on competitive climate (Ames and Ames, 1984; Nerstad et al., 2013; Černe et al., 2014a,b) as well as the literature on leaders’ power construal (Fousiani and Wisse, 2022). Importantly, this research highlights that in the context of AI, employee attitudes and actual use of AI are influenced not merely by the technology’s features, but also by the broader organizational climate and leadership style. This contributes to our understanding of the multifaceted factors that shape AI acceptance in the workplace (Schepman and Rodway, 2023). Taken together, these results contribute to a more holistic understanding of the interplay between competitive climate, leader behavior, and AI use, offering broader implications for future research. In addition, our research has several practical implications. When operating in competitive climates, organizations seeking to foster AI adoption must acknowledge the pivotal role of leadership. For the improvement of AI acceptance and integration, organizational policymakers and top management should promote a perspective among leaders where they construe their power as responsibility to support and empower their employees. For instance, organizations can create training programs and interventions to equip leaders with the necessary skills and knowledge to adopt a responsible leadership approach. By prioritizing employees’ psychological safety and providing a supportive environment through responsible leadership, leaders can mitigate the potential threats associated with competitive climates and cultivate a more favorable view of AI.

### Strengths, limitations and future directions

This research carries several strengths as well as limitations. Theoretical strengths lie in the comprehensive exploration of competitive climate, leaders’ power construal, and AI acceptance, providing insight into their intricate interplay. Additionally, the study presents a novel perspective by considering how leader power construal influences the relationship between competitive climate and AI acceptance. Our study has various methodological strengths as well, as it uses a multi-method approach, combining a field study and an experiment, enhancing the robustness of the findings. However, there are some limitations too. The field study’s findings, which indicate that the competitive climate has no significant effect on AI attitudes, raise questions about the strength of this association. Furthermore, the measure we used to assess leader’s power construal in Study 1 was relatively short, although well-established (de Wit et al., 2017), and might not capture its full complexity. Moreover, the unexpected negative main effects of competitive climate on AI acceptance in the experiment require further investigation to align with the findings of the field study. Importantly, the sample size of Study 2 was relatively small, which may have influenced the robustness of the obtained results. Finally, this research lacks the inclusion of mediators, consequently rendering it unable to provide explanatory mechanisms for the observed effects. Future research should include potential mediating variables to explain the obtained results. For example, future research should examine the mediating role of experienced threat or challenge of employees (i.e., the extent to which demands exceed or outweigh one’s resources; Blascovich and Tomaka, 1996; Blascovich, 2008) in the prediction of AI acceptance within competitive work climates, particularly when leaders construe their power either as responsibility or as opportunity. These limitations provide valuable pointers for future research, offering opportunities to enhance our understanding of the intricate dynamics between competitive climates, leadership styles, and AI acceptance.

Overall, it is important to highlight the dynamic nature of AI, which can impact the timeliness and applicability of research findings. In particular, the rapid advancement and widespread adoption of AI within organizations can significantly shorten the shelf life of research results. For instance, only a few months ago, AI was relatively unfamiliar and novel in professional settings, but today, a substantial number of employees have seamlessly integrated it into their daily work routines. This may influence the significance and timeliness of even recent research findings in this field, and researchers should take it into account when investigating the antecedents of AI acceptance. Furthermore, AI is inherently multifaceted, encompassing a wide range of methodologies and applications. When investigating attitudes towards AI and its integration into professional contexts, it is crucial to recognize the diverse forms and applications of AI, as well as the specific industries it is involved in. These specifics can profoundly shape employees’ attitudes towards AI as some may perceive AI as a precious tool that enhances their work, while others may view it as a potential competitor that threatens their job security (Frank et al., 2019). Additionally, it is vital to acknowledge that the unique characteristics of the companies and organizations under examination can significantly influence employees’ AI attitudes and AI use (Yuan et al., 2022). While this research controlled for the impact of organizational sector in the relationships between the focal variables, more comprehensive efforts are needed to account for the influence of other variables in the examined relationships.

## Conclusion

Investigating the incorporation of AI in organizations is pivotal to understand how to optimally steer work performance and innovation, and our research contributes to understanding the factors relating to AI acceptance. Although the results of the field study and the experiment were inconsistent regarding the effect of competitive climate on AI acceptance, they consistently demonstrated the important role that leader power construal plays in shaping AI attitudes and AI use within competitive climates. Practically, organizations can encourage AI adoption within competitive climates by prioritizing responsible leadership, prioritizing the interests of employees, and providing leaders with the necessary training. In contrast, in order to promote AI acceptance, organizations should discourage the presence of opportunistic leadership within competitive environments.

## Data availability statement

The datasets presented in this study can be found in online repositories. The names of the repository/repositories and accession number(s) can be found at: https://osf.io/dau3m/?view_only=fa173ca739dc476d9a0a11828162eee6.

## Ethics statement

The studies involving humans were approved by Ethics Committee Board, University of Groningen, Netherlands. The studies were conducted in accordance with the local legislation and institutional requirements. The participants provided their written informed consent to participate in this study.

## Author contributions

KF: Conceptualization, Data curation, Formal analysis, Investigation, Methodology, Project administration, Software, Supervision, Writing – original draft, Funding acquisition, Writing – review & editing. GM: Data curation, Formal analysis, Methodology, Writing – original draft, Writing – review & editing. PM: Conceptualization, Methodology, Writing – review & editing. KJ: Conceptualization, Funding acquisition, Methodology, Writing – review & editing.

## Footnotes

^1^RMSEA (Root Mean Square Error of Approximation) assesses how well the model fits the observed data, ranging from 0 to 1 with lower values indicating better model fit; CFI (Comparative Fit Index) analyses the specified model’s fit by examining the data and the hypothesized model. Values range from 0 to 1, with larger values indicating better fit; SRMR (Standardized Root Mean Square Residual) is a measure of the average difference between observed correlation and the model-implied correlation. Values range from 0 to 1, with lower values suggesting better model fit.

^2^Our initial sample comprised 380 participants. For the manipulation of the leader’s power construal, we incorporated high vs. low power as responsibility conditions, high vs. low power as opportunity conditions, and a control condition. However, the results became less straightforward and interpretable with the inclusion of all these conditions in the analysis. To simplify and achieve more interpretable outcomes, we opted to exclude the low power as responsibility, low power as opportunity, and control conditions from the main analyses. Consequently, the main analyses only encompassed the conditions of high power as opportunity vs. high power as responsibility, as these conditions are more pertinent to our research hypotheses. The data for all conditions are publicly available on the Open Science Framework.

## References

[ref1] Aboelmaged M. G. (2014). Predicting e-readiness at firm-level: an analysis of technological, organizational and environmental (TOE) effects on e-maintenance readiness in manufacturing firms. Int. J. Inf. Manag. 34, 639–651. doi: 10.1016/j.ijinfomgt.2014.05.002

[ref2] Alsheibani S. Cheung Y. Messom C. (2018). Artificial intelligence adoption: AI-readiness at firm-level. Twenty-Second Pacific Asia Conference on Information Systems, Yokohama, Japan.

[ref3] Amabile T. M. (2020). Creativity, artificial intelligence, and a world of surprises. Acad. Manag. Discov. 6, 351–354. doi: 10.5465/amd.2019.0075

[ref4] Ames C. Ames R. (1984). Goal structures and motivation. Elem. Sch. J. 85, 39–52. doi: 10.1086/461390

[ref5] Bandura A. (1977). Self-efficacy: toward a unifying theory of behavioral change. Psychol. Rev. 84, 191–215. doi: 10.1037/0033-295X.84.2.191 847061

[ref6] Bhargava A. Bester M. Bolton L. (2021). Employees’ perceptions of the implementation of robotics, artificial intelligence, and automation (RAIA) on job satisfaction, job security, and employability. J. Technol. Behav. Sci. 6, 106–113. doi: 10.1007/s41347-020-00153-8

[ref7] Blascovich J. (2008). “Challenge and threat” in Handbook of approach and avoidance motivation. ed. Elliot A. J. (London, United Kingdom: Psychology Press), 431–445.

[ref8] Blascovich J. Tomaka J. (1996). “The biopsychosocial model of arousal regulation” in Advances in experimental social psychology. ed. Zanna M. P., vol. 28 (London, United Kingdom: Academic Press), 1–51.

[ref9] Broadbent E. Kerse N. Peri K. Robinson H. Jayawardena C. Kuo T.. (2016). Benefits and problems of health-care robots in aged care settings: a comparison trial. Australas. J. Ageing 35, 23–29. doi: 10.1111/ajag.12190 26364706

[ref10] Brougham D. Haar J. (2018). Smart technology, artificial intelligence, robotics, and algorithms (STARA): employees’ perceptions of our future workplace. J. Manag. Organ. 24, 239–257. doi: 10.1017/jmo.2016.55

[ref11] Brown C. Reich M. Ulman L. Nakata Y. (1998). Work and pay in the United States and Japan. Oxford, United Kingdom: Oxford University Press.

[ref003] Černe M. Dimovski V. Marič M. Penger S. Škerlavaj M. (2014a). Congruence of leader self-perceptions and follower perceptions of authentic leadership: Understanding what authentic leadership is and how it enhances employees’ job satisfaction. Aust. J. Manag. 39, 453–471. doi: 10.1177/0312896213503665

[ref004] Černe M. Nerstad C. G. Dysvik A. Škerlavaj M. (2014b). What goes around comes around: Knowledge hiding, perceived motivational climate, and creativity. Acad. Manage. J. 57, 172–192. doi: 10.5465/amj.2012.0122

[ref12] Chui M. Manyika J. Miremadi M. (2015). Four fundamentals of workplace automation. Seattle, Washington, USA: McKinsey Quarterly.

[ref13] Chung K.-C. (2014). Gender, culture and determinants of behavioural intents to adopt mobile commerce among the Y generation in transition economies: evidence from Kazakhstan. Behav. Inform. Technol. 33, 743–756. doi: 10.1080/0144929X.2013.805243

[ref14] Chung M. Ko E. Joung H. Kim S. J. (2020). Chatbot e-service and customer satisfaction regarding luxury brands. J. Bus. Res. 117, 587–595. doi: 10.1016/j.jbusres.2018.10.004

[ref15] Clark M. A. Michel J. S. Zhdanova L. Pui S. Y. Baltes B. B. (2016). All work and no play? A meta-analytic examination of the correlates and outcomes of workaholism. J. Manag. 42, 1836–1873. doi: 10.1177/0149206314522301

[ref16] Compeau D. R. Higgins C. A. (1995). Computer self-efficacy: development of a measure and initial test. MIS Q. 19:189. doi: 10.2307/249688

[ref17] De Jonge K. M. M. Rietzschel E. F. Nijstad B. A. (in press). “Working with the ideas of others” in The research handbook on workplace creativity. eds. Goncalo J. Katz J.

[ref18] De Wit F. Scheepers D. Ellemers N. Sassenberg K. Scholl A. (2017). Whether power holders construe their power as responsibility or opportunity influences their tendency to take advice from others. J. Organ. Behav. 38, 923–949. doi: 10.1002/job.2171

[ref001] Draxler F. Werner A. Lehmann F. Hoppe M. Schmidt A. Buschek D.. (2023). The AI ghostwriter effect: Users do not perceive ownership of AI-generated text but self-declare as authors. arXiv preprint. arXiv:2303.03283.

[ref19] Enholm I. M. Papagiannidis E. Mikalef P. Krogstie J. (2022). Artificial intelligence and business value: a literature review. Inf. Syst. Front. 24, 1709–1734. doi: 10.1007/s10796-021-10186-w

[ref20] Fast E. Horvitz E. (2017). Long-term trends in the public perception of artificial intelligence. Proceedings of the AAAI Conference on Artificial Intelligence 31. doi: 10.1609/aaai.v31i1.10635

[ref21] Fletcher T. D. Major D. A. Davis D. D. (2008). The interactive relationship of competitive climate and trait competitiveness with workplace attitudes, stress, and performance. J. Organ. Behav. 29, 899–922. Available at: http://www.jstor.org/stable/30163356

[ref002] Fousiani K. de Jonge K. Michelakis G. (2022). Having no negotiation power does not matter as long as you can think creatively: The moderating role of age. Int. J. Confl. Manag. 33, 956–990. doi: 10.1108/IJCMA-05-2022-0086

[ref22] Fousiani K. Wisse B. (2022). Effects of leaders’ power construal on leader-member exchange: the moderating role of competitive climate at work. J. Leadersh. Organ. Stud. 29, 306–324. doi: 10.1177/15480518221075229

[ref23] Frank M. R. Autor D. Bessen J. E. Brynjolfsson E. Cebrian M. Deming D. J.. (2019). Toward understanding the impact of artificial intelligence on labor. PNAS 116, 6531–6539. doi: 10.1073/pnas.1900949116 30910965 PMC6452673

[ref24] Hughes C. Robert L. Frady K. Arroyos A. (2019). “Artificial intelligence, employee engagement, fairness, and job outcomes” in Managing technology and middle-and low-skilled employees. eds. Hughes I. C. Robert L. Frady K. Arroyos A. (Leeds, UK: Emerald Publishing Limited), 61–68.

[ref25] Jones A. P. James L. R. (1979). Psychological climate: dimensions and relationships of individual and aggregated work environment perceptions. Organ. Behav. Hum. Perform. 23, 201–250. doi: 10.1016/0030-5073(79)90056-4

[ref26] Kaplan A. Haenlein M. (2019). Siri, Siri, in my hand: Who’s the fairest in the land? On the interpretations, illustrations, and implications of artificial intelligence. Bus. Horiz. 62, 15–25. doi: 10.1016/j.bushor.2018.08.004

[ref27] Kim J.-Y. Heo W. (2022). Artificial intelligence video interviewing for employment: perspectives from applicants, companies, developer and academicians. Inf. Technol. People 35, 861–878. doi: 10.1108/ITP-04-2019-0173

[ref28] Kirkpatrick K. (2017). AI in contact centers. Commun. ACM 60, 18–19. doi: 10.1145/3105442

[ref29] Kohn A. (1992). No contest: the case against competition. New York: Houghton Mifflin Harcourt.

[ref30] Li J. J. Bonn M. A. Ye B. H. (2019). Hotel employee’s artificial intelligence and robotics awareness and its impact on turnover intention: the moderating roles of perceived organizational support and competitive psychological climate. Tour. Manag. 73, 172–181. doi: 10.1016/j.tourman.2019.02.006

[ref31] McClure P. K. (2018). “You’re fired” says the robot: the rise of automation in the workplace, technophobes, and fears of unemployment. Soc. Sci. Comput. Rev. 36, 139–156. doi: 10.1177/0894439317698637

[ref32] Mikalef P. Gupta M. (2021). Artificial intelligence capability: conceptualization, measurement calibration, and empirical study on its impact on organizational creativity and firm performance. Inf. Manag. 58:103434. doi: 10.1016/j.im.2021.103434

[ref006] Muthén L. K. Muthén B. O. (2017). Mplus: Statistical analysis with latent variables: User’s Guide (Version 8). Los Angeles, CA: Authors.

[ref007] Nerstad C. G. L. Roberts G. C. Richardsen A. M. (2013). Achieving success at work: Development and validation of the Motivational Climate at Work Questionnaire (MCWQ). J. Appl. Soc. Psychol. 43, 2231–2250. doi: 10.1111/jasp.12174

[ref33] Oliveira T. Martins M. F. (2011). Literature review of information technology adoption models at firm level. Electron. J. Inf. Syst. Eval. 14, 110–121.

[ref34] Pullig C. Maxham J. G. III Hair J. F. Jr. (2002). Salesforce automation systems: an exploratory examination of organizational factors associated with effective implementation and salesforce productivity. J. Bus. Res. 55, 401–415. doi: 10.1016/S0148-2963(00)00159-4

[ref35] Pumplun L. Tauchert C. Heidt M. (2019). A new organizational chassis for artificial intelligence – Exploring organizational readiness factors. In Proceedings of the 27th European Conference on Information Systems (ECIS), Stockholm & Uppsala, Sweden, June 8–14, 2019. ISBN 978-1-7336325-0-8 Research Papers. Available at: https://aisel.aisnet.org/ecis2019_rp/106.

[ref36] Raelin J. A. (2003). Creating leaderful organizations: how to bring out leadership in everyone. San Francisco: Berrett-Koehler.

[ref37] Raisch S. Krakowski S. (2021). Artificial intelligence and management: the automation–augmentation paradox. Acad. Manag. Rev. 46, 192–210. doi: 10.5465/amr.2018.0072

[ref39] Rosen L. D. Weil M. M. (1995). Computer anxiety: a cross-cultural comparison of university students in ten countries. Comput. Hum. Behav. 11, 45–64. doi: 10.1016/0747-5632(94)00021-9

[ref40] Sassenberg K. Ellemers N. Scheepers D. (2012). The attraction of social power: the influence of construing power as opportunity versus responsibility. J. Exp. Soc. Psychol. 48, 550–555. doi: 10.1016/j.jesp.2011.11.008

[ref009] Sassenberg K. Ellemers N. Scheepers D. Scholl A. (2014). “Power corrupts revisited: The role of construal of power as opportunity or responsibility,” in Power, politics, and paranoia: Why people are suspicious of their leaders. Eds. Van Prooijen J.-W. Van Lange P. A. M. (United Kingdom: Cambridge University Press), 73–87.

[ref41] Scheepers D. De Wit F. Ellemers N. Sassenberg K. (2012). Social power makes the heart work more efficiently: evidence from cardiovascular markers of challenge and threat. J. Exp. Soc. Psychol. 48, 371–374. doi: 10.1016/j.jesp.2011.06.014

[ref42] Schepman A. Rodway P. (2020). Initial validation of the general attitudes towards artificial intelligence scale. Comput. Human Behav. Rep. 1:100014. doi: 10.1016/j.chbr.2020.100014 PMC7231759 34235291

[ref43] Schepman A. Rodway P. (2023). The general attitudes towards artificial intelligence scale (GAAIS): confirmatory validation and associations with personality, corporate distrust, and general trust. Int. J. Human Comput. Interact. 39, 2724–2741. doi: 10.1080/10447318.2022.2085400

[ref44] Scholl A. Sassenberg K. Scheepers D. Ellemers N. De Wit F. (2017). A matter of focus: power-holders feel more responsible after adopting a cognitive other-focus, rather than a self-focus. Br. J. Soc. Psychol. 56, 89–102. doi: 10.1111/bjso.12177 27900793

[ref005] Scholl A. Sassenberg K. Ellemers N. Scheepers D. T. De Wit F. (2018). Highly identified powerholders feel responsible: The interplay between social identification and social power in groups. Br. J. Soc. Psychol. 57, 112–129. doi: 10.1111/bjso.12225 28983928

[ref45] Schrock W. A. Hughes D. E. Fu F. Q. Richards K. A. Jones E. (2016). Better together: trait competitiveness and competitive psychological climate as antecedents of salesperson organizational commitment and sales performance. Mark. Lett. 27, 351–360. doi: 10.1007/s11002-014-9329-7

[ref46] Schwab K. (2017). The fourth industrial revolution. Crown: World Economic Forum.

[ref47] Syam N. Sharma A. (2018). Waiting for a sales renaissance in the fourth industrial revolution: machine learning and artificial intelligence in sales research and practice. Ind. Mark. Manag. 69, 135–146. doi: 10.1016/j.indmarman.2017.12.019

[ref48] Vargas R. Yurova Y. V. Ruppel C. P. Tworoger L. C. Greenwood R. (2018). Individual adoption of HR analytics: a fine grained view of the early stages leading to adoption. Int. J. Hum. Resour. Manag. 29, 3046–3067. doi: 10.1080/09585192.2018.1446181

[ref49] Venkatesh V. Morris M. G. Davis G. B. Davis F. D. (2003). User acceptance of information technology: toward a unified view. MIS Q. 27, 425–478. doi: 10.2307/30036540

[ref50] Vinchon F. Lubart T. Bartolotta S. Gironnay V. Botella M. Bourgeois-Bougrine S.. (2023). Artificial intelligence and creativity: a manifesto for collaboration. J. Creat. Behav. 57, 472–484. doi: 10.1002/jocb.597

[ref51] Wamba-Taguimdje S.-L. Fosso Wamba S. Kala Kamdjoug J. R. Tchatchouang Wanko C. E. (2020). Influence of artificial intelligence (AI) on firm performance: the business value of AI-based transformation projects. Bus. Process. Manag. J. 26, 1893–1924. doi: 10.1108/BPMJ-10-2019-0411

[ref52] Wayne S. J. Ferris G. R. (1990). Influence tactics, affect, and exchange quality in supervisor-subordinate interactions: a laboratory experiment and field study. J. Appl. Psychol. 75, 487–499. doi: 10.1037/0021-9010.75.5.487

[ref53] Wilkens U. (2020). Artificial intelligence in the workplace – a double-edged sword. Int. J. Inf. Learn. Technol. 37, 253–265. doi: 10.1108/IJILT-02-2020-0022

[ref54] Wisse B. Rus D. (2012). Leader self-concept and self-interested behavior: the moderating role of power. J. Pers. Psychol. 11, 40–48. doi: 10.1027/1866-5888/a000054

[ref55] Wisse B. Rus D. Keller A. C. Sleebos E. (2019). “Fear of losing power corrupts those who wield it”: the combined effects of leader fear of losing power and competitive climate on leader self-serving behavior. Eur. J. Work Organ. Psy. 28, 742–755. doi: 10.1080/1359432X.2019.1635584

[ref56] Wright S. A. Schultz A. E. (2018). The rising tide of artificial intelligence and business automation: developing an ethical framework. Bus. Horiz. 61, 823–832. doi: 10.1016/j.bushor.2018.07.001

[ref57] Ye B. H. Tung V. W. S. Li J. J. Zhu H. (2020). Leader humility, team humility and employee creative performance: the moderating roles of task dependence and competitive climate. Tour. Manag. 81:104170. doi: 10.1016/j.tourman.2020.104170

[ref58] Yu X. Xu S. Ashton M. (2023). Antecedents and outcomes of artificial intelligence adoption and application in the workplace: the socio-technical system theory perspective. Inf. Technol. People 36, 454–474. doi: 10.1108/ITP-04-2021-0254

[ref59] Yuan P. Fabian F. Ni L. Yunyang H. Maolin Y. (2022). The adoption of artificial intelligence in employee recruitment: the influence of contextual factors. Int. J. Hum. Resour. Manag. 33, 1125–1147. doi: 10.1080/09585192.2021.1879206

