# Supplementary material for: Competitive organizational climate and artificial intelligence (AI) acceptance: the moderating role of leaders’ power construal
Source: Front Psychol. 2024 Mar 25;15:1359164. doi: 10.3389/fpsyg.2024.1359164 (PMC11003519; doi:10.3389/fpsyg.2024.1359164)
Supplement: Supplementary file 1 [file Data_Sheet_1.docx]

**Online Supplemental Material**

**Study 1 - Field Study**

***Measures***

**Competitive Climate** (Nerstad et al., 2013) – Measured at T1

Please answer the following questions about how things work at your department/work group.

1. In my department/work group, there exists a competitive rivalry among the employees.
2. In my department/work group, work accomplishments are measured based on comparisons with the accomplishments of coworkers.
3. In my department/work group, rivalry between employees is encouraged.
4. In my department/work group, internal competition is encouraged to attain the best possible results.
5. In my department/work group, only those employees who achieve the best results/accomplishments are set up as examples.
6. In my department/work group, one is encouraged to perform optimally to achieve monetary rewards.
7. In my department/work group, an individual’s accomplishments are compared with those of other colleagues.
8. In my department/work group, it is important to achieve better than others.

(1 = *Strongly disagree*; 7 = *Strongly agree*)

**Power Construal (Opportunity vs. Responsibility)** (De Wit et al., 2017) - Measured at T2

Please indicate the extent to which you agree or disagree with each of the following statements.

In my work, my supervisor tends to see his/her power in terms of…

1. The *opportunity* that it gives him/her to tell subordinates what to do without having to ask them what they actually want to do.
2. The *responsibility* it gives him/her towards his/her subordinates and their needs.
3. The *opportunity* it gives him/her to make their own decisions without having to think about their subordinates’ desires or needs.
4. The *obligations* it gives him/her towards his/her subordinates (e.g., take care of things that need to be done).
5. The *opportunity* it gives him/her to achieve goals that he/she finds important for himself/herself rather than for his/her subordinates.
6. The *responsibility* to ensure that important goals of his/her subordinates are met.

(1 = *Not at all true*; 7 = *Absolutely true*)

**Positive Attitude towards Artificial Intelligence Scale (**Schepman & Rodway, 2020) Measured at T2 and T3

We are interested in your attitudes towards Artificial Intelligence. By Artificial Intelligence we mean devices that can perform tasks that would usually require human intelligence.

Please note that these can be computers, robots or other hardware devices, possibly augmented with sensors or cameras, etc. Please complete the following scale, indicating your response to each item. There are no right or wrong answers. We are interested in your personal views.

1. For routine transactions, I would rather interact with an artificially intelligent system than with a human.
2. Artificial Intelligence can provide new economic opportunities for this country.
3. Organizations use Artificial Intelligence unethically. (R)
4. Artificially intelligent systems can help people feel happier.
5. I am impressed by what Artificial Intelligence can do.
6. I think artificially intelligent systems make many errors. (R)
7. I am interested in using artificially intelligent systems in my daily life.
8. I find Artificial Intelligence sinister. (R)
9. Artificial Intelligence might take control of people. (R)
10. I think Artificial Intelligence is dangerous. (R)
11. Artificial Intelligence can have positive impacts on people’s wellbeing.
12. Artificial Intelligence is exciting.
13. An artificially intelligent agent would be better than an employee in many routine jobs.
14. There are many beneficial applications of Artificial Intelligence.
15. I shiver with discomfort when I think about future uses of Artificial Intelligence. (R)
16. Artificially intelligent systems can perform better than humans.
17. Much of society will benefit from a future full of Artificial Intelligence.
18. I would like to use Artificial Intelligence in my own job.
19. People like me will suffer if Artificial Intelligence is used more and more. (R)
20. Artificial Intelligence is used to spy on people. (R)

(1 = *Strongly disagree*; 7 = *Strongly agree*)

R= score reversed items, indicating negative attitudes towards artificial intelligence.

**Actual use of Artificial Intelligence Scale** (adapted from Draxler et al., 2023) Measured at T2 and T3

How often do you use Artificial Intelligence to manage conflicts in the workplace?

I use Artificial Intelligence to find creative solutions to conflict situations at work

I use Artificial Intelligence to find new perspectives to resolve conflict in the workplace

I use Artificial Intelligence to find novel arguments in conflict situations at work

I use Artificial Intelligence to combine and improve perspectives in conflict resolution in the workplace

(1 = *Very rarely /Almost never*; 7 = *Very often*)

**Study 2 - Experiment**

***Scenario Instructions***

Below you will read a description of a situation at work. Read the story carefully and try to place yourself in that situation. It is important that you try to immerse yourself into that situation fully. Try to imagine what you would feel, think and do if you were in that situation.

Imagine that you are a member of the marketing department for PBF, a large multinational company in the fast-moving consumer goods (FMCG) sector. The team in which you work consists of 17 team members and also has a boss - a supervisor named Bill.

***Power Construal Manipulation***

**High Power as Opportunity.** Your supervisor, Bill, is a person who sees his power as a great opportunity to influence others to his own advantage and as a chance to tell others what to do. He is the type of leader who feels that he can focus on the opportunities to achieve goals that he finds important for himself. For instance, in a recent conversation with him, Bill told you that he is using the possibility that his position as a supervisor gives him to make decisions that determine his own outcomes as well as those of his subordinates (ranging from the tasks to be performed, to the trainings to attend, and the bonus one is eligible for). Bill indeed always makes use of this opportunity. His motto is: “Power gives you the chance to look out for your own interest and you should always use that option”.

**High Power as Responsibility.** Your supervisor, Bill, is a person who sees his power as a great responsibility towards others and as an obligation towards other people to take care of things that need to be done. He is the type of leader who feels responsible for ensuring that important group goals are met. For instance, in a recent conversation with him, Bill told you that he is well aware of the responsibility that his position as a supervisor gives him to make decisions that have important consequences for himself but also for his subordinates (ranging from the tasks to be performed, to the trainings to attend, and the bonus one is eligible for). Bill indeed always takes care of these commitments. His motto is: “Power gives you the duty to look out for other people’s interest and you should always do that”.

**Manipulation Checks - Power Construal (Fousiani & Wisse, 2022)**

Now that you have a clear picture in your mind regarding your supervisor and the organization you are working for, we would like to ask you some questions about it. Based on what you have just read, please indicate to what extent you agree with the following statements.

1. Bill uses the power that comes with his supervisory position as an opportunity to influence his subordinates to his own advantage. (1 = *Not at all*, 7 = *To a great extent*)
2. Bill uses the power that comes with his supervisory position as a means to fulfill his responsibility towards his subordinates. (1 = *Not at all*; 7 = *To a great extent*)
3. It is not specified in the text how Bill sees the power that comes with his supervisory position.

(1 = *Strongly disagree*; 7 = *Strongly agree*)

***Competitive Climate Manipulation***

**Competitive Climate - High.** Notably, the climate in your company is very competitive. Only the top achievers have the chance to get rewarded and promoted. Moreover, these top achievers are often singled out as heroic examples of excellent performance. Internal competition among employees is not only encouraged but actively promoted and individuals’ performance gets ranked in comparison to others. Everyone in your company (including your colleagues) is aware of this climate. Therefore, there is substantial competitive rivalry among employees at all levels. As an employee, you are very much aware of the competitive climate and the high rivalry at your work, as you experience it every single day.

**Competitive Climate - Low.** Notably, the climate in your company is not very competitive. Most employees have a chance to get rewarded and promoted. Moreover, individual employees are not singled out as heroic examples of excellent performance. Internal competition among employees is not only discouraged but actively disapproved of and individuals’ performance is judged on its own merits, regardless of how others are performing. Everyone in your company (including your colleagues) is aware of this climate. Therefore, there is no competitive rivalry among employees at any levels. As an employee, you are very much aware of the non-competitive climate and the absence of rivalry at your work, as you experience it every single day.**Manipulation Checks - Competitive Climate** (Fousiani & Wisse, 2022)

1. The climate in this company is competitive.
2. In this company rivalry among employees is encouraged.
3. In this company an individual’s accomplishments are compared with those of other colleagues.

(1 = *Strongly disagree*; 7 = *Strongly agree*)

**Positive Attitude towards Artificial Intelligence - General** (adapted from Draxler et al., 2023)

Considering the depiction of your organization and your supervisor, Bill in the scenario that you read, how probable is the occurrence of the following?

1. How likely is Artificial Intelligence to be funded in your organization, as described in the scenario you read?
2. How likely is Artificial Intelligence to be helpful in your organization, as described in the scenario you read?

(1 = *Very unlikely*; 7 = *Very likely*)

***Vignette***

**“Working hours per week”:** In the company you are working at, a standard working week is 36 hours. Your supervisor, Bill, wants you to work fewer hours per day but more days per week. You disagree. You want to work more hours per day but fewer days per week.

How will you approach this disagreement/conflict with your supervisor, Bill? As you answer the following questions, please keep in mind the description of your company climate and the specific characteristics of your supervisor, Bill.

**Positive Attitudes towards Artificial Intelligence for Managing the Conflict at Hand** (adapted from Draxler et al., 2023)

*How helpful do you consider Artificial Intelligence to be in disagreements/conflicts like this one, between yourself and your supervisor, Bill?*

Artificial Intelligence can be a positive force in managing this conflict with my supervisor, Bill.

Artificial Intelligence can be helpful if used in this situation.

*(1= Strongly disagree, 7 = Strongly agree)*

**Likelihood of Using Artificial Intelligence to Manage the Conflict at Hand** (adapted from Draxler et al., 2023)

*How likely would you use Artificial Intelligence to manage this disagreement/conflict with your supervisor, Bill?*

I would use Artificial Intelligence to find creative solutions to this disagreement/conflict with my supervisor, Bill.

I would use Artificial Intelligence to combine and improve perspectives in conflict resolution in situations like the one described between myself and my supervisor, Bill.

*(1= Very unlikely, 7 = Very likely)*

**Additional Vignette, not Included in the Analysis**

**“Deadlines of deliverables”:** You need to set the deadlines for the deliverables of a project on which both of you (you and your supervisor, Bill), have been working jointly. You disagree with one another on the deadlines that are set for the deliverables. Your supervisor, Bill, wants to submit the project deliverables earlier whereas you want to submit later.
How will you approach this disagreement/conflict with your supervisor, Bill? Again, as you answer the following questions, please keep in mind the description of your company climate and the specific characteristics of your supervisor, Bill.
